# A Highly Diverse Olenekian Brachiopod Fauna from the Nanpanjiang Basin, South China, and Its Implications for the Early Triassic Biotic Recovery

**DOI:** 10.3390/biology12040622

**Published:** 2023-04-19

**Authors:** Huiting Wu, Yang Zhang, Anfeng Chen, Thomas L. Stubbs

**Affiliations:** 1 School of Geoscience and Surveying Engineering, China University of Mining and Technology (Beijing), Beijing 100083, China; ht_wu415@163.com; 2 State Key Laboratory of Palaeobiology and Stratigraphy, Nanjing Institute of Geology and Palaeontology, CAS, Nanjing 210008, China; 3 School of Earth Sciences and Resources, China University of Geosciences (Beijing), Beijing 100083, China; af_chen18@163.com; 4 School of Earth Sciences, University of Bristol, Bristol BS1 5QD, UK; tom.stubbs@bristol.ac.uk

**Keywords:** Early Triassic, brachiopod, biotic recovery, benthos, Nanpanjiang basin, Datuguan section

## Abstract

**Simple Summary:**

Brachiopods have been thought to be in very low diversity in the Early Triassic for a long time. There are only several Olenekian brachiopod fauna reported worldwide, all of which are in very low diversity. This paper reports the most diverse Olenekian brachiopod fauna so far, containing 14 species with nine genera. Among them, three new species are proposed, and six genera are found in the Early Triassic for the first time. This diverse fauna indicates that the diversity of Olenekian brachiopod fauna has been underestimated. Based on precise age constrained by conodont biostratigraphy and quantitative data of brachiopod, it can be inferred that brachiopod recovery in the studied section occurred in the latest Spathian rather than the Smithian when the environment started to ameliorate. Global brachiopod data also indicates that the initial recovery of brachiopods happened in the Spathian.

**Abstract:**

As one of the predominant benthic organisms in the Palaeozoic, brachiopod was largely eliminated in the Permian–Triassic boundary mass extinction, and then highly diversified in the Middle Triassic. Since fossil data from the Early Triassic are rarely reported, the recovery patterns of Early Triassic brachiopods remain unclear. This study documents a well-preserved fauna that is the most diverse Olenekian brachiopod fauna so far (age constrained by conodont biostratigraphy) from the Datuguan section of ramp facies in South China. This fauna is composed of 14 species within nine genera, including six genera (*Hirsutella*, *Sulcatinella*, *Paradoxothyris*, *Dioristella*, *Neoretzia* and *Isocrania*) found in the Early Triassic for the first time and three new species, including *Paradoxothyris flatus* sp. nov., *Hirsutella sulcata* sp. nov. and *Sulcatinella elongata* sp. nov. The Datuguan fauna indicates that the diversity of Olenekian brachiopod fauna has been underestimated, which can be caused by a combination of reduced habitats (in geographic size and sedimentary type) compared with the end-Permian, great bed thickness making it difficult to find fossils and most species in the fauna having low abundance. Based on the faunal change in the Datuguan section and environmental changes in South China, it can be inferred that brachiopod recovery in the studied section occurred in the latest Spathian rather than the Smithian when the environment started to ameliorate. Global brachiopod data also indicates that the initial recovery of brachiopods happened in the Spathian, and many genera that widely occurred in the Middle or Late Triassic had originated in the Olenekian.

## 1. Introduction

The Permian–Triassic boundary mass extinction (PTBME) event greatly disrupted marine ecosystems, which transformed from ‘Palaeozoic-type’ to ‘Mesozoic- and Cenozoic-type’ fauna [1,2,3,4,5,6,7]. The hostile environmental conditions (e.g., deadly high temperature [8]; anoxia event [9]) caused by volcanism were proposed to trigger this major biological crisis, and then these persistent environmental disasters and significantly decreased diversity limited the biotic recovery process in the post-extinction interval. Brachiopods were largely eliminated [10], but this gave way to a new evolutionary stage, and brachiopods subsequently evolved modest taxonomic, morphological, functional and ecological diversity in the Mesozoic and Cenozoic [11,12]. New ecomorphologies appeared, with changes from reclining and anchoring to pedicle-fixing ecologies [11], and changes from brachial ridges and spiralia to loops, spiralia and crura in the mineralised lophophore supports [12]. Through the whole Triassic, the Athyridida, Spiriferinida, Terebatulida and Rhynchonellida, which are characterised by the pedicle fixing type and loops, spiralia or crura supports, are the dominant brachiopod orders.

In the post-extinction interval, Induan brachiopod fauna is mostly reported from South China, characterised by the transient Permian holdovers (e.g., *Paryphella*, *Fusichonetes*, *Prelissorhynchia*, *Paracrurithyris*) [13,14,15], lingulids [16,17] and a few newcomers (e.g., *Meishanorhynchia*, *Lichuanorelloides*) [18,19]. In the Olenekian, brachiopod fauna is rarely reported (Idaho, western USA [20,21]; Qilian Area, north-western China [22]; Primorye, Russia [23]; Romania [24]; and Tibet, China [25]), and have very low diversity in most of these areas. Up until now, glimpses into the evolutionary dynamics of Early Triassic brachiopods (excluding Permian holdovers) show very low diversity and few occurrences. If this is true, what constraints (such as global warming, anoxia, and biotic interaction) brachiopod diversity and recovery patterns in the Early Triassic?

To investigate the early evolution of Triassic brachiopods, this study reports an Olenekian brachiopod fauna from the Datuguan section, Nanpanjiang Basin, southern Guizhou Province, South China. It is currently the most diverse fauna when compared to global contemporaries. This paper also provides several brachiopod fossil data models to outline how the main drivers (e.g., extinction event, environmental factors, sampling bias) influenced brachiopod diversity and recovery in the Early Triassic.

## 2. Geological Settings and Age

South China (especially the Yangtze Block part) is one of the few regions in the world yielding successive Lower to Middle Triassic strata, spanning a continuum of depositional environments from the nearshore clastic shelf, carbonate platform, offshore clastic shelf, ramp, isolated carbonate platform and basin [26]. Abundant trace fossils [27], and abundant and diversified marine organisms have been reported from these strata (e.g., gastropods [28,29,30]; brachiopods [18,31]; ammonoids [32,33]; ostracods [34]; bivalves [35]; and foraminifers [36]).

**Figure 1:**
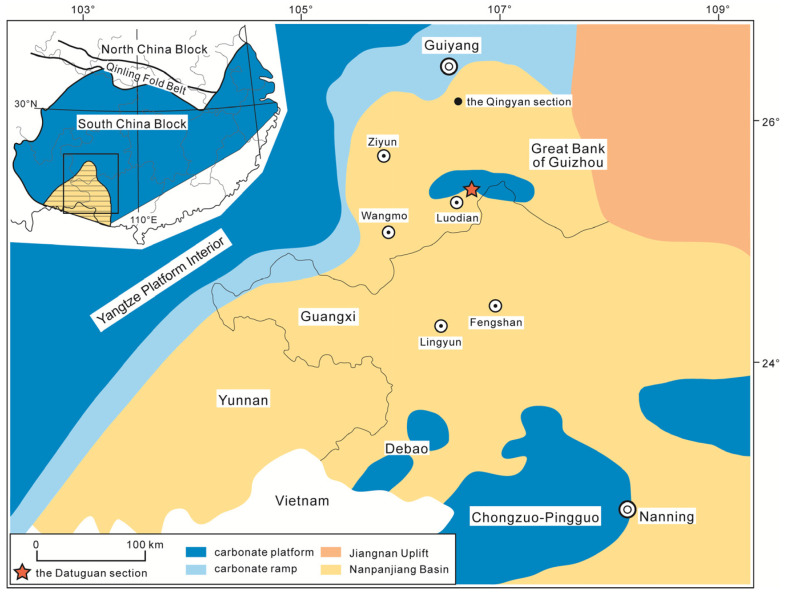
Early Triassic palaeogeographical map of Nanpanjiang Basin, South China (modified after [37]), showing the location of the Datuguan section and Qingyan section.

**Figure 2:**
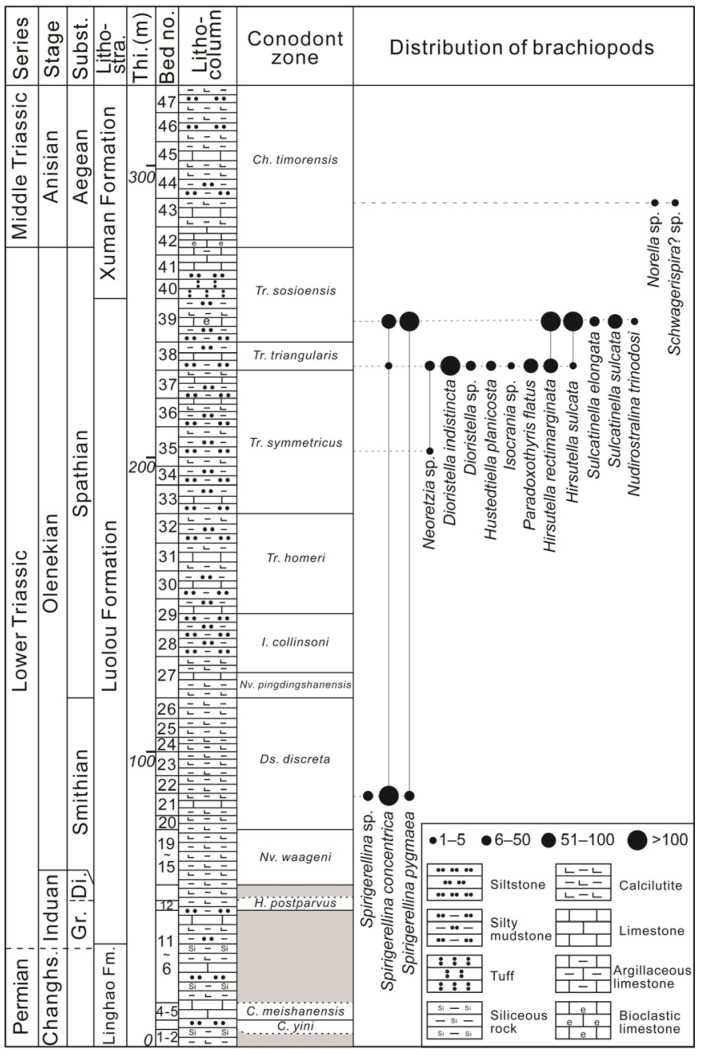
Distribution of brachiopods and zonations of conodont from the Linghao, Luolou and Xuman formations in the Datuguan section. Conodont data are from [38]. Subst., Substage; Lithostra., Lithostratigraphy; Thi., Thickness; Changhs., Changhsingian; Gr., Griesbachian; Di., Dienerian; Fm., Formation; *C*., *Clarkina*; *H*., *Hindeodus*; *Nv*., *Novispathodus*; *Ds*., *Discretella*; *I*., *Icriospathodus*; *Tr*., *Triassospathodus*, *Ch*., *Chiosella*.

The Datuguan section is located 120 km south of Guiyang City and 5 km north of Luodian County. The section occurs on the southern ramp (below the storm wave base) of the Great Guizhou Bank from the Changhsingian (Late Permian) to Middle Triassic (Figure 1). At the Datuguan section, the upper Changhsingian strata belong to the Linghao Formation, which mainly contains dark grey thin-bedded siliceous mudstone, yellow-green thin-bedded calcareous mudstone and grey thick-bedded micritic limestone. The Induan and Olenekian strata are represented by the Luolou Formation, which is characterised by fawn medium-bedded calcareous mudstone and greyish-green medium-bedded siltstone, intercalated with dark grey medium-bedded micritic limestone. The Anisian strata are represented by the Xuman Formation, which mainly includes greyish-green tuff (only at the bottom), greyish-green medium-bedded siltstone, fawn medium-bedded calcareous mudstone, intercalated with dark grey medium-bedded micritic limestone (Figure 2).

Strata of the Datuguan section from the Changhsingian (uppermost Permian) to the Anisian (lower Middle Triassic) are precisely defined by successive conodont biostratigraphy [38]. Based on the first occurrence of *Novispathodus waageni*, *Nv. pingdingshanensis* and *Chiosella timorensis*, the Induan–Olenekian boundary, the Smithian–Spathian boundary and the Olenekian–Anisian boundary are placed at the bottom of Bed 16, Bed 27 and the middle of Bed 42, namely the bottom of the Luolou Formation, the middle of the Luolou Formation and the bottom of the Xuman Formation (Figure 2).

## 3. Materials and Methods

In total, 1583 complete brachiopod specimens were collected from the Luolou Formation (Beds 21, 35, 38 and 39) and basal part of the Xuman Formation (Bed 46). All the specimens (accessible upon request from the corresponding author) are and will be permanently deposited in the Laboratory of Palaeontology, College of Geoscience and Surveying Engineering, China University of Mining and Technology, Beijing, China, with the prefixes LD.

To estimate completeness of sampling, a rarefaction analysis was applied and conducted using PAST (Palaeontological Statistics [39]). In order to investigate the changes to craniformean and rhychonelliformean brachiopod diversity and abundance from the Wuchiapingian (Late Permian) to the Anisian (Middle Triassic), brachiopod genera and occurrence data were collected from the Paleobiology Database (PBDB) (http://paleobiodb.org, up to 30 June 2022) and Treatise on Invertebrate Palaeontology Part H: Brachiopoda, Volume 2–6 [40,41,42,43]. When counting the occurrence frequency of a genus, specimens occurred in the same section were counted as one occurrence. Since this paper mainly focused on the ‘Mesozoic-type’ brachiopod, the occurrence data of Lingulida were not collected. The data downloaded from the PBDB used the following parameters: time intervals = Changhsingian and Rhaetian, and Taxon = brachiopoda. All brachiopod data have been checked and revised according to the most recently accepted classifications, and records with uncertainty were not included herein.

## 4. Results

**Figure 3:**
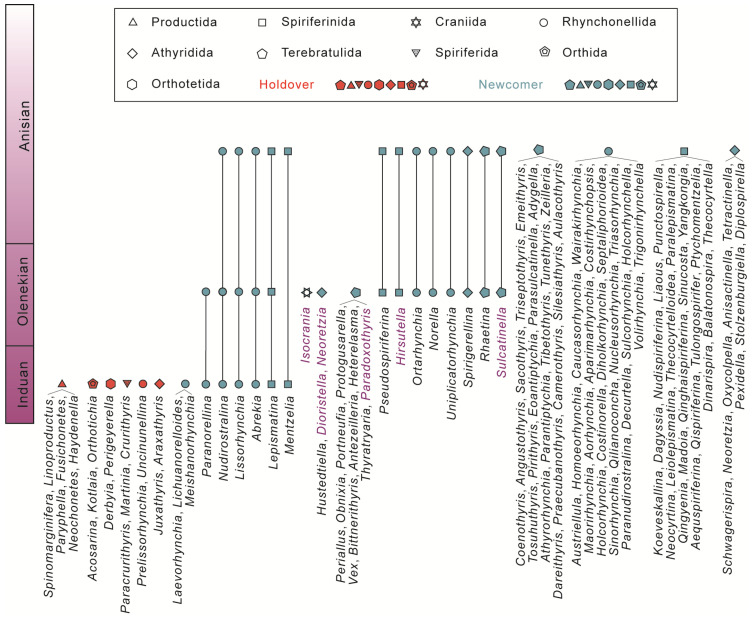
Occurrences of brachiopod genera (Lingulida excluded) worldwide during the Early and Middle Triassic based on data from the Datuguan section and PBDB. Genera shown in purple are described from the Olenekian for the first time in this paper.

A total of 16 species in 11 genera are recognised in the Datuguan brachiopod fauna (Figure 2), including three newly proposed species, *Hirsutella sulcata* sp. nov., *Paradoxothyris flatus* sp. nov. and *Sulcatinella elongata* sp. nov. Among the 11 genera of the Datuguan fauna, six of them are found in the Early Triassic for the first time (*Hirsutella*, *Sulcatinella*, *Paradoxothyris*, *Dioristella*, *Neoretzia* and *Isocrania*), and occupied nearly a quarter of brachiopod genera reported in the Olenekian (Figure 3).

**Figure 4:**
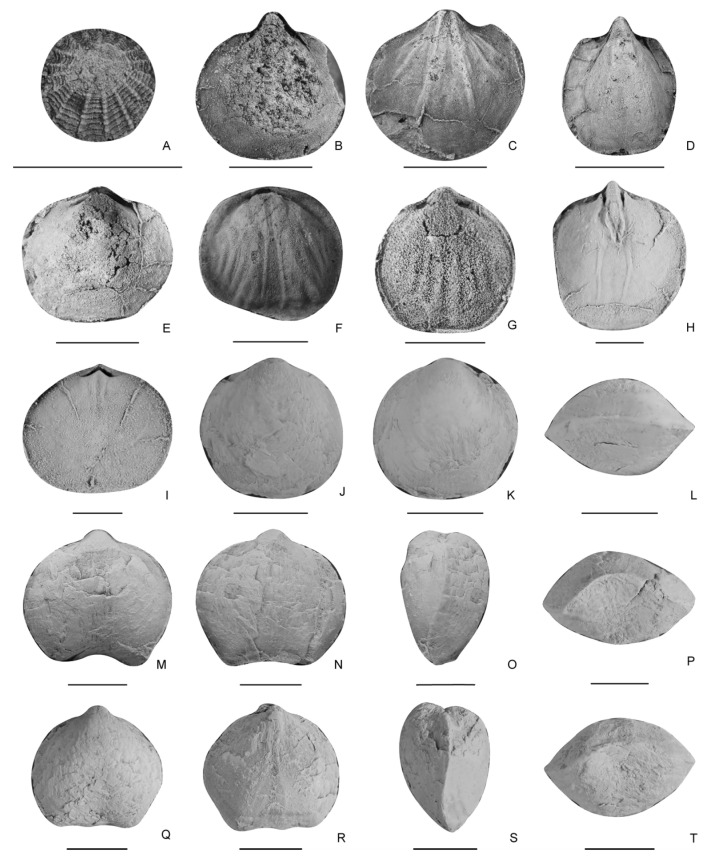
(**A**), *Isocrania* sp., ventral valve, LD380288. (**B**–**E**), *Dioristella indistincta*, (**B**–**D**), internal moulds of ventral valve, LD380119, LD380219, LD380396; I, an internal mould of a dorsal valve, LD380413. (**F**,**G**), *Dioristella* sp., internal moulds of dorsal valves, LD380200, LD380141. (**H**–**L**), *Spirigerellina concentrica*, (**H**), an internal mould of a ventral valve, LD385533; (**I**), an internal mould of a dorsal valve, LD385605; (**J**–**L**), ventral, dorsal and anterior views of a conjoined shell, LD385352. (**M**–**T**), *Spirigerellina pygmaea*, ventral, dorsal, lateral and anterior views of two conjoined shells, LD385207, LD385260. In (**A**–**L**), scale bar = 2 mm, in (**M**–**T**), scale bar = 5 mm.

**Figure 5:**
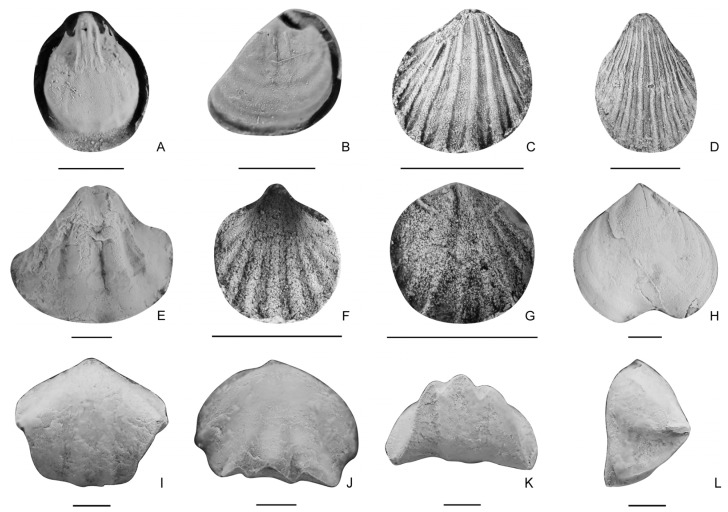
(**A**,**B**), *Spirigerellina* sp., (**A**), an internal mould of a ventral valve, LD215553; (**B**), an internal mould of a dorsal valve, LD215554. (**C**,**D**), *Hustedtiella planicosta*, (**C**), an internal mould of a dorsal valve, LD380056; (**D**), an internal mould of a ventral valve, LD380272. (**E**), *Schwagerispira*? sp., a ventral valve, LD435623. (**F**,**G**), *Neoretzia* sp., (**F**), an external mould of a ventral valve, LD380267; (**G**), an internal mould of a dorsal valve, LD380172. (**H**), *Norella* sp., a ventral valve, LD435517. (**I**–**L**), *Nudirostralina trinodosi*, ventral, dorsal, anterior and lateral views of a conjoined shell, LD381185. Scale bar = 2 mm.

**Figure 6:**
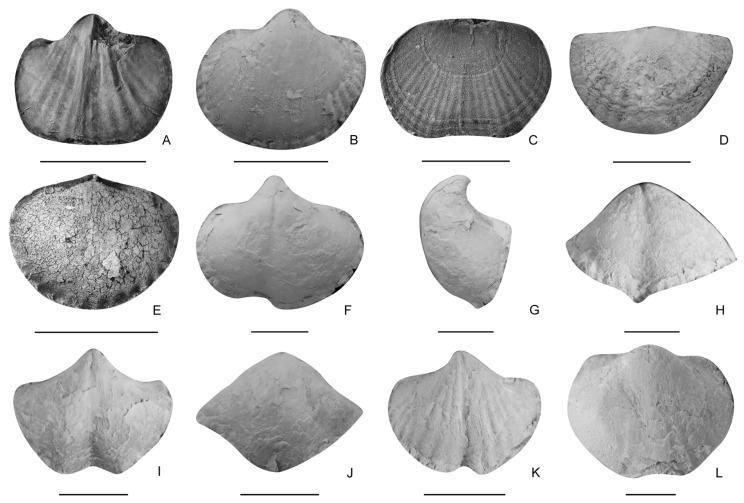
(**A**–**E**), *Hirsutella rectimarginata*, (**A**), an internal mould of a ventral valve, LD380429; (**B**), a ventral valve, LD383507; (**C**), an external mould of a dorsal valve, LD380440; (**D**), a dorsal valve, LD384020; (**E**), an internal mould of a dorsal valve, LD380253. (**F**–**L**), *Hirsutella sulcata* sp. nov., (**F**–**K**), ventral valves, (**F**), LD383719, (**I**), LD384237, (**K**), LD384281; (**G**,**H**), lateral and anterior views of (**F**); (**J**), anterior view of (**I**); (**L**), a dorsal valve, LD383526. Scale bar = 5 mm.

**Figure 7:**
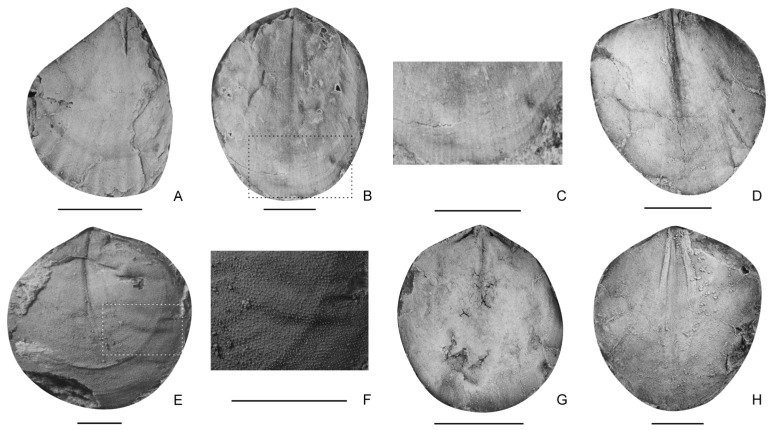
*Paradoxothyris flatus* sp. nov., (**A**,**B**), internal moulds of ventral valves, LD380016, LD380303, (**C**), enlarged area of (**B**); (**D**–**H**), internal moulds of dorsal valves, LD380100, LD380419, LD380125, LD380204, (**F**), enlarged area of (**E**). Scale bar = 2 mm.

**Figure 8:**
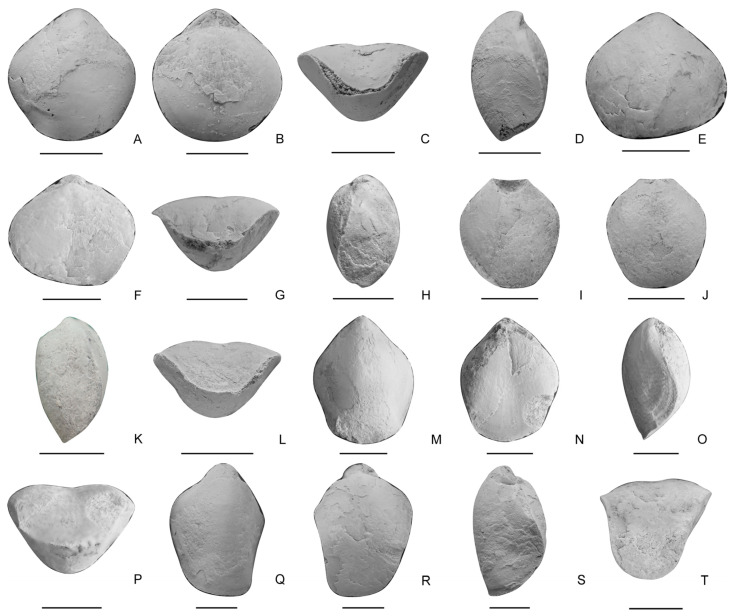
(**A**–**H**), *Sulcatinella sulcata*, ventral, dorsal, lateral and anterior views of two conjoined shells, LD384756, LD384786. (**I**–**T**), *Sulcatinella elongata* sp. nov., ventral, dorsal, lateral and anterior views of a conjoined shell, LD384755, LD384907, LD384867. Scale bar = 5 mm.

There are only several Olenekian brachiopod fauna found worldwide so far (Table 1), mainly including those reported from western North America [20,21], the Balkan region [24], the Far East of Russia [23], north-western China [22], and Tibet [25]. Compared with those fauna, the Datuguan brachiopod fauna shows the highest richness (14 species in nine genera in the Olenekian) (Table 1, Figure 4, Figure 5, Figure 6, Figure 7 and Figure 8).

## 5. Systematic Palaeontology

Order Spiriferinida Ivanova, 1972a [44]
Suborder Cyrtinidina Carter and Johnson in Carter, et al., 1994 [45]
Superfamily Suessioidea Waagen, 1883 [46]
Family Bittnerulidae Schuchert, 1929 [47]
Subfamily Hirsutellinae Xu and Liu in Yang, et al., 1983 [22]
Genus *Hirsutella* Cooper and Muir-Wood, 1951 [48]
*Hirsutella sulcata* sp. nov. (Figure 6F–L)

Derivation of name. In reference to the prominent sulcus in the ventral valve.

Diagnosis. Rounded subpentagonal to semicircular outline, distinct ventral sulcus with an extended median tongue.

Type specimen. Holotype, a ventral valve (LD384281, Figure 6K); paratype, a ventral valve (LD383719, Figure 6F).

Other material. A ventral valve (LD384237), a dorsal valve (LD383526).

Description. Shell medium in size for genus, 7.68–13.5 mm in length and 9.12–16.6 mm in width (12 specimens measured), rounded subpentagonal to semicircular in outline; maximum width at about midvalve; hinge slightly shorter than shell width; cardinal extremities rounded. Ventral valve moderately convex; umbo highly elevated and inflated, moderately incurved; beak narrow and pointed; sulcus beginning from umbo or midvalve, widening and deepening anteriorly, with a median tongue distinctly extended. Dorsal valve moderately convex, umbo slightly over hinge; lateral slopes strongly inclined, fold wide and elevated, not well demarcated from lateral slopes. External surface covered with costae, obscure at umbonal region, and even invisible when the surface layer of the shell is peeled off.

Remarks. The present species is similar to *Hirsutella extraruga* (Yang and Yin in Yang, et al., 1962) [49] in shell outline and lateral profile, but it has a more distinct sulcus and an extended median tongue occasionally developed in the ventral valve. *Hirsutella hirsuta* (Alberti, 1864) [50] is similar to the present species in shell outline and development of ventral sulcus, but differs by having a more elevated ventral beak. The current species is similar to *Sinucosta bifucata* Sun and Shi, 1985 [51] from the upper Triassic of Yunnan, China, in the rounded subpentagonal outline and moderately developed costae, but the former has a wider hinge, coarser costae and more distinct fold and sulcus. The Datuguan specimens resemble *Mentzelia subspherica* Yang and Xu, 1966 [52] from the Anisian of Guizhou, south China, in semicircular outline and sulcus beginning from beak, but the latter has more costae than most of the Datuguan specimens and developed spondylium. It is similar to *Dagyssia multicostata* (Yang and Xu, 1966) [52] from Qinghai, China, in the similar outline and feebly developed costae, but the latter has less developed sulcus and fold and more number of costae.

Distribution. Olenekian; China.

Order Terebratulida Waagen, 1883 [46]
Suborder Terebratulidina Waagen, 1883 [46]
Superfamily Dielasmatoidea Schuchert, 1913 [53]
Family Angustothyrididae Dagys, 1972b [54]
Genus *Paradoxothyris* Xu, 1978 [55]
*Paradoxothyris flatus* sp. nov. (Figure 7)

Derivation of name. In reference to the low convexity of both valves.

Diagnosis. Rounded lateral and anterior margins, variably developed median ridges on both valves, both valves slightly convex or nearly flattened, sometimes with regularly distributed costellae.

Type specimen. Holotype, an internal mould of a ventral valve (LD380303, Figure 7B); paratype, an internal mould of a dorsal valve (LD380419, Figure 7E).

Other material. An internal mould of ventral valve (LD380016), and three internal moulds of dorsal valves (LD380100, LD380125, LD380204).

Description. Shell small to medium in size for genus, 2.92–9.44 mm in length and 2.25–8.39 mm in width (16 specimens measured), elongated suboval in outline; maximum width at middle to the anterior part of the shell. Ventral valve slightly convex to nearly flat; maximum convexity at umbo; posterior margin V-shaped, lateral and anterior margins very rounded; sulcus absent; interior with a weak median ridge beginning from beak and extending to one-fifth to a half of shell length, and absent in some specimens. Dorsal valve nearly flat; margins curved; fold absent; interior with a median ridge beginning from beak, and extending to about one-sixth to one-half of shell length; sockets long and narrow, and inner socket ridges thin and diverging at an angle of about 105°. Shell punctate; external surface sometimes ornamented with fine and dense costellae at the middle to the anterior part of the shell.

Remarks. The present species is similar to *Paradoxothyris cyclis* Xu, 1978 [55], *Paradoxothyris sangkaensis* (Jin, et al., 1979) [56] and *Paradoxothyris pentagona* (Jin, et al., 1979) [56] in the absence of a fold and sulcus, but differs in having a much less convex ventral valve and almost flat dorsal valve.

Distribution. Olenekian; China.

**Figure 9:**
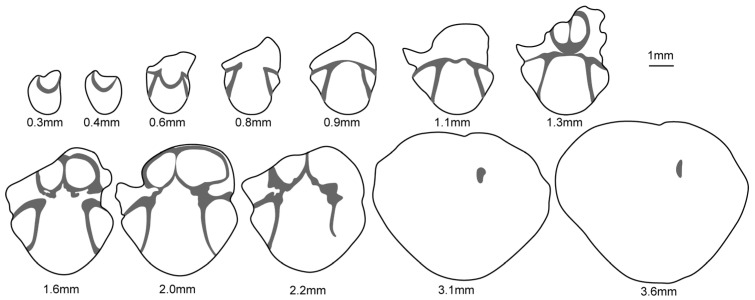
Serial sections of *Sulcatinella elongata* sp. nov., LD384907.

Family Dielasmatidae Schuchert, 1913 [53]
Subfamily Dielasmatinae Schuchert, 1913 [53]
Genus *Sulcatinella* Dagys, 1974 [57]
*Sulcatinella elongata* sp. nov. (Figure 8I–T and Figure 9)

Derivation of name. In reference to the elongated outline.

Diagnosis. Elongated rhombic to subpentagonal outline, distinctly inclined lateral slopes of ventral valve, strong unisulcate anterior commissure.

Type specimen. Holotype, a conjoined shell (LD384867, Figure 8Q–T), paratype, a conjoined shell (LD384907, Figure 8M–P).

Other material. A conjoined shell (LD384755).

Description. Shell medium to large in size for genus, 9.16–16 mm in length and 7.19–12.5 mm in width (12 specimens measured), elongated rhombic to subpentagonal in outline; lateral commissure moderately to strongly incurved towards the dorsal side, anterior commissure strong unisulcate. Ventral valve moderately convex; beak slightly curved; posterior margin V-shaped, lateral and anterior margins straight to slightly curved; fold elevated, beginning from the umbonal region and widening anteriorly; lateral slopes flattened to slightly convex, distinctly inclined towards dorsal valve; interior with distinct and short pedicle collar; dental plates slightly diverging at an angle of about 30° (Figure 9). Dorsal valve slightly to moderately convex; sulcus strong, originating from umbo, distinctly widening and deepening anteriorly, strongly bending towards ventral valve at anterior part; interior with distinct and large crural bases, inner hinge plates converging at an angle of about 65° to form a V-shaped septalium, connected with median septum, septalium and septum disappear at about the same time (Figure 9).

**Figure 10:**
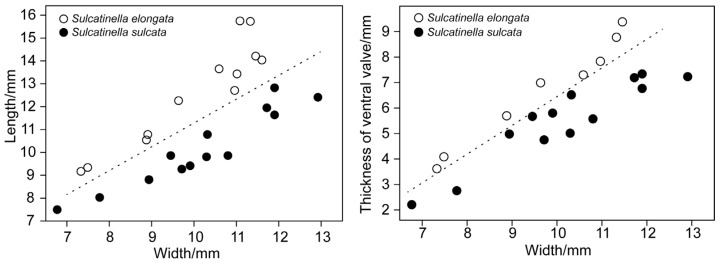
Graph of shell length to shell width and thickness of ventral valve to width of *S. sulcata* and *S. elongata* from the studied section.

Remarks. Shell length, width and thickness of ventral valve of *S. sulcata* and *S. elongata* specimens from the studied section are measured. The length-to-width ratio is adopted to represent the shell outline, and the thickness of ventral valve-to-width ratio is used to represent the inclination of lateral slopes of ventral valve. It is shown that the present species differs from *S. sulcata* by having a more elongated outline and more strongly inclined lateral slopes of ventral valve (Figure 10). It is similar to *S. incrassata* by Grădinaru and Gaetani [24] in the elongated subpentagonal outline and shell convexity, but differs by having a much more curved lateral commissure and wider dorsal sulcus. The Datuguan specimens resemble *Angustothyris qingyanensis* Guo et al., 2020 [31] from the Anisian in having an elongated outline, unisulcate anterior commissure and smooth shells, but differ by having distinctly developed dental plates and strongly declined lateral slopes of ventral valve.

Distribution. Olenekian; China.

## 6. Discussion

### 6.1. The Hidden Diversity in the Early Triassic

**Figure 11:**
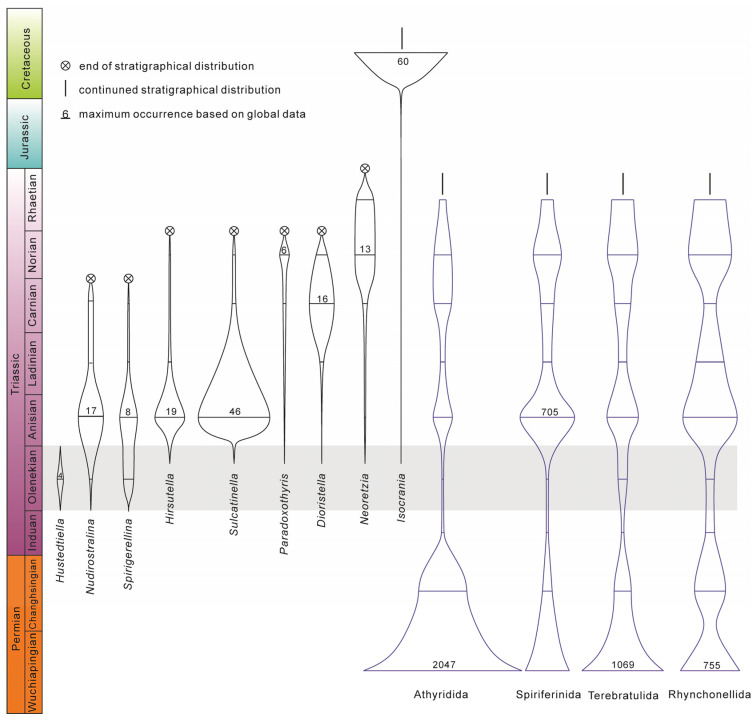
Stratigraphic ranges and occurrences of the Datuguan brachiopod genera (shown by black lines) and four brachiopod orders (shown by purple lines). Numbers above lines represent the highest occurrence frequency of genera.

Olenekian brachiopod fauna was thought to be in very low diversity for a long time (as is shown in Table 1, wherein the species richness index was chosen to measure diversity); however, this newly discovered Luodian fauna shows a very high diversity (14 species in nine genera) and thus indicates a very likely hidden brachiopod diversity in the Early Triassic. Pietsch, et al., [58] referred to the hidden echinoid diversity of the Early Triassic. Massive diversity losses during the extinction event, coupled with hidden diversity in the recovery, result in evolutionary bottlenecks. If we examine the diversity changes of craniformean and rhynchonelliformean brachiopods from the Lopingian to Triassic, an evolutionary bottleneck existed in all the brachiopod orders Rhynchonellida, Spiriferinida, Terebratulida and Athyridida, which all have their lowest diversity in the Early Triassic [12,40,41,42,43] (Figure 3 and Figure 11). Evident evolutionary bottlenecks widely occurred in marine organisms during the Early Triassic (e.g., radiolarians, foraminiferous, ammonoids [6]), and are a typical evolutionary pattern for the transitional interval between the mass extinction and subsequent completed ecosystem recovery. Generally, there are several parameters which can result in the phenomenon of hidden diversity, including the reduced habitat in geographic size and sedimentary type, taxa abundance and great bed thickness.

During the Early Triassic, persistently deteriorated ocean environments made habitats hostile for marine organisms [8,9,59,60], especially for the benthos, and there were only a few ‘habitable zones’ in some specific environments [61,62,63]. In this case, the habitats of craniformean and rhynchonelliformean brachiopods were significantly reduced in the Early Triassic. The largely shrunken habitats in the Early Triassic oceanic environment would clearly reduce brachiopod abundance and the probability of fossil preservation and discovery, and could lead to considerable underestimation of Early Triassic brachiopod abundance and diversity.

**Figure 12:**
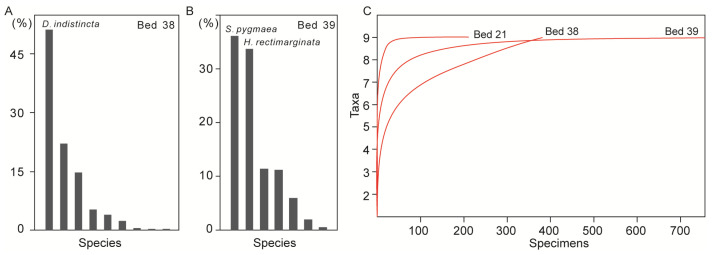
(**A**,**B**), frequency distribution of brachiopods from bed 38 (nine species) and bed 39 (seven species) in the Datuguan section; (**C**), results of rarefaction analysis of brachiopod data from three main beds yielding brachiopods in the Datuguan section. *D*: *Dioristella*; *S*: *Spirigerellina*; *H*: *Hirsutella*.

How does taxa abundance affect diversity? We chose the Datuguan brachiopod fauna (Spathian, this study) and Jianzishan brachiopod fauna (Dienerian [19]) as examples to demonstrate the abundance model. As shown in Figure 12A,B, the brachiopod communities of Beds 38 and 39 are both characterised by one or two dominant species, and more than half of the species have very low abundance (less than 20 in Bed 38). The Jianzishan brachiopod fauna, which contains *Lichuanorelloides lichuanensis* (212 specimens), *Lissorhynchia* sp. (86 specimens) and *Crurithyris* sp. (eight specimens) [19], is a typical Induan-type fauna with an absolute dominant taxon. In this case, Early Triassic brachiopod fauna, which contains a dominant species and many low-abundance species, is very likely to underestimate diversity due to inadequate sampling.

As for bed thickness, it is reasonable to speculate that fossil sampling is more difficult in thicker strata of the same duration. In South China, the Early Triassic strata are much thicker than the end-Permian strata. Most of the Early–Middle Triassic brachiopod fauna in South China are reported from ramp environments (the Yinkeng Formation [18]; the Xinyuan Formation [64]; the Daye Formation [19]; and the Qingyan Formation [30]), where much thinner strata are yielded than in shallow water settings. Weak hydrodynamic conditions in the deeper water environments could help to preserve fossils, and thinner strata would clearly increase the chance of fossil discovery.

To summarise, Early Triassic marine ecosystems, which existed between the collapse of Palaeozoic-type ecosystems and the final reconstruction of Mesozoic-type ecosystems, are characterised by high dominance and low evenness. The great loss of diversity (extinction event) [65,66] and hostile environments [8,9] led to the high dominance of certain taxa (disasters, opportunists, newcomers) within these fauna, which further limited the abundance of other species. This pattern is one of the most important features of Early Triassic marine ecosystems, and could be one of the main reasons for substantially underestimated diversity. We examined the occurrences of nine genera found in the Olenekian from the studied section, and discovered that they all have the fewest records in the Olenekian, and six of them were first reported in the Early Triassic (Figure 11). In this case, adequate sampling (1583 complete specimens, Figure 12C) and condensed strata (the Luolou Formation in the ramp) should minimise the effect of sampling biases on brachiopod faunal diversity.

### 6.2. Brachiopod Recovery Pattern in the Post-Extinction Interval

In the post-extinction interval, the Induan (especially Griesbachian) brachiopod fauna was mainly characterised by holdovers (26 genera), and the newcomers, which included a few genera of Rhynchonellida (seven genera) and Spiriferinida (two genera) (Figure 3). If we exclude the holdovers in the earliest Induan fauna, the Smithian and most Induan brachiopod fauna generally have very low diversity [18,19,25,67], which should be categorised in the ‘survival stage’ of the overall recovery process.

**Figure 13:**
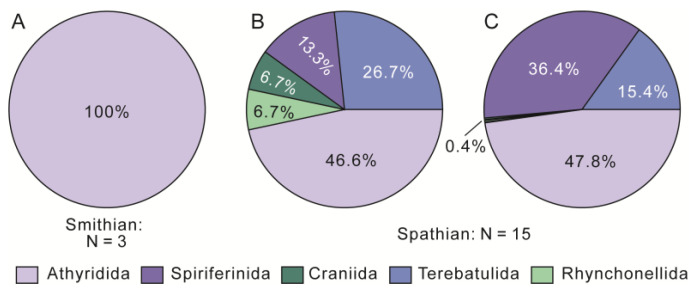
Composition of the Datuguan brachiopod fauna. (**A**,**B**) are based on species amount data, and (**C**) is based on specimen data. N, number of species.

Most of these previously reported Olenekian fauna are mainly composed of Rhynchonellida, Terebratulida, Spiriferinida and Athyridida, except for that from Dobrogea (Romania), which only contains Rhynchonellida [24]. The faunal composition at the order level is consistent with the statistical data of brachiopod genera in the Triassic [43], and represents the initial stage of brachiopod evolution in the post-extinction interval. The Datuguan brachiopod fauna only includes Athyridida (three species within one genus) in the Smithian, and is dominated by Athyridida, Spiriferinida and Terebatulida in both species (13 species within nine genera), and specimen counts in the Spathian (Figure 13). This suggests that the brachiopod recovery occurred in the Spathian rather than Smithian, which is consistent with the global biotic recovery event for this substage [68,69,70].

The fossil horizons (Beds 38 and 39) of the Datuguan section yielding abundant brachiopods are restricted to the *Triassospathodus triangularis* and *Tr. sosioensis* conodont zones (Figure 2), which indicate a latest Spathian Age [71,72,73]. According to [8], the lethally hot temperatures started to fall, and a cooling event occurred in the latest Spathian (upper part of *Tr. homeri* Zone). Based on the evidence from pyrite framboids, the redox condition in ramp settings also started to improve from an anoxic–lower dysoxic to upper dysoxic–oxic environment in the latest Spathian of the Qingyan section (northern margin of Nanpanjiang Basin) (upper part of *Tr. homeri* Zone [9]) (Figure 1). As for the benthos, the Datuguan brachiopod fauna started to diversify in this improved habitat in the latest Spathian. The deteriorated environments (e.g., deadly temperatures, anoxia) in low latitudinal areas since the latest Permian evidently started to return to normal conditions after nearly five million years [8,9,59,60], and the reconstruction of the Triassic marine ecosystem truly began in the latest Spathian.

Unexpectedly, based on global data from online databases and published works of literature, the recovery rate of Olenekian (especially Spathian) brachiopod fauna (28 genera) has been substantially undervalued. Brachiopods showed high diversity in the late Olenekian, which is three times that in the Induan, and more than one-third of that in the Anisian (84 genera), and is therefore indicative of an initial recovery in the Spathian. It is noteworthy that some brachiopod genera, which have their maximum occurrences in the Middle or Late Triassic, already started to appear in the Olenekian age (Figure 11).

In addition, the fact that most contemporaneous brachiopod fauna are reported from the Balkan region, Primorye, the Qilianshan region, the Nanpanjiang basin and Idaho, indicates that the Palaeo-Tethys Sea region and the western margin of North America provided the most important habitats for brachiopods in the Spathian Age. These areas were actually the most hostile habitats during the Permian–Triassic boundary mass extinction event [59,74,75]. Environmental amelioration in tropical regions indicates an overall improvement of global oceanic environments, which might then have given rise to the subsequent overall recovery during the Middle Triassic.

## 7. Conclusions

1. A Olenekian brachiopod fauna which is the most diverse one so far, is reported in this study. It contains 14 species in nine genera, among which *Hirsutella*, *Sulcatinella*, *Paradoxothyris*, *Dioristella*, *Neoretzia* and *Isocrania* are found in the Early Triassic for the first time, and three species are newly proposed;
2. Brachiopod abundance and diversity data indicated that brachiopod recovery in the studied section happened in the latest Spathian when the environmental condition (deadly temperatures and anoxia) started to ameliorate;
3. One of the reasons that brachiopod was widely considered to be in very low abundance in the Early Triassic was the phenomenon of hidden diversity. It could be caused by the decrease of habitat, low taxa abundance and great thickness of strata.

## Figures and Tables

**Table 1:** Main Olenekian brachiopod fauna worldwide, fauna with only one or two species not included herein.

Location	Genus/ Species	Order	Age	Reference
Primorye, Russia	6/6	Rhynchonellida, Terebratulida, Spiriferinida, Athyridida	Olenekian	[23]
Qilian Area, China	4/10	Rhynchonellida, Terebratulida, Athyridida	Olenekian	[22]
Tibet, China	3/3	Rhynchonellida, Terebratulida, Athyridida	Smithian	[25]
Idaho, USA	4/5	Rhynchonellida, Terebratulida, Spiriferinida	Spathian	[20,21]
Dobrogea, Romania	3/3	Rhynchonellida	Spathian	[24]
Guizhou, China	9/14	Rhynchonellida, Terebratulida, Spiriferinida, Athyridida, Craniida	Olenekian	This study

## Data Availability

Data available on request from authors.
